# The Impact of Point-of-Care Testing for Group A Streptococcal Pharyngitis on Antibiotic Prescribing and Patient Health Outcomes in Outpatient Care: A Systematic Review and Meta-analysis of Randomized Controlled Trials

**DOI:** 10.1093/ofid/ofaf407

**Published:** 2025-07-09

**Authors:** Ann-Sophie Mägdefrau, Carolin Kathner-Schaffert, Anni Matthes, Jutta Bleidorn, Robby Markwart

**Affiliations:** Jena University Hospital, Institute of General Practice and Family Medicine, Friedrich Schiller University Jena, Jena, Germany; InfectoGnostics Research Campus Jena, Jena, Germany; Jena University Hospital, Institute of General Practice and Family Medicine, Friedrich Schiller University Jena, Jena, Germany; Jena University Hospital, Institute of General Practice and Family Medicine, Friedrich Schiller University Jena, Jena, Germany; InfectoGnostics Research Campus Jena, Jena, Germany; Jena University Hospital, Institute of General Practice and Family Medicine, Friedrich Schiller University Jena, Jena, Germany; Jena University Hospital, Institute of General Practice and Family Medicine, Friedrich Schiller University Jena, Jena, Germany; InfectoGnostics Research Campus Jena, Jena, Germany

**Keywords:** antibiotic prescribing, group A β-hemolytic *Streptococcus*, pharyngitis, point-of-care tests, rapid diagnostic tests

## Abstract

**Background:**

Point-of-care testing (POCT) for group A β-hemolytic *Streptococcus* (StrepA) allows for rapid testing for streptococcal infection in patients with signs of pharyngitis. We conducted a systematic review and meta-analysis of the impact of StrepA POCTs on antibiotic prescribing and health outcomes in patients with signs of pharyngitis in outpatient care.

**Methods:**

Medline, Web of Science, Scopus, and Cochrane Central Register of Controlled Trials were searched for randomized controlled trials (RCTs; January 2000–January 2025). Random-effects models were used to calculate pooled risk ratios (RRs) with 95% confidence intervals (CIs) for summary effect sizes.

**Results:**

From 15 097 unique records, we identified 8 eligible RCTs comparing the use of StrepA POCTs with standard care. The use of StrepA POCTs reduced the number of antibiotics prescribed by 38% (RR, 0.62 [95% CI, .51–.77]; *P* < .001). In studies with StrepA POCTs as the sole intervention, antibiotic prescribing was reduced by 41% (RR, 0.59 [95% CI, .44–.78]; *P* < .001; 5 RCTs). The reduction in antibiotic prescribing was observed in children (RR, 0.56 [95% CI, .39–.81]; *P* = .002; 4 RCTs) and adults (RR, 0.57 [95% CI, .39–.85]; *P* = .006; 2 RCTs). The number of follow-up healthcare visits did not differ between StrepA POCT and standard care (RR, 0.56 [95% CI, .29–1.09]; *P* = .086, 3 RCTs). There were no differences between point estimates of individual RCTs for other patient health outcomes, such as days until pain resolution and days of school/work missed.

**Conclusions:**

The use of StrepA POCTs in children and adults with signs of pharyngitis likely reduces the number of antibiotics prescribed without compromising patient health outcomes.

Pharyngitis, also referred to as sore throat or tonsillitis, is the inflammation of the pharynx primarily caused by viral and bacterial infections. Group A β-hemolytic *Streptococcus* (StrepA), also known as *Streptococcus pyogenes*, is the most prevalent bacterium associated with pharyngitis [1]. It accounts for 15%–40% of pharyngitis cases in children and 5%–15% in adults [2, 3]. StrepA pharyngitis is of significant public health importance due to its high incidence (∼616 million cases per year [4]) and its potential to cause severe complications, including long-term sequelae like rheumatic heart disease, as well as outbreaks in settings like schools and healthcare facilities.

Although the majority of pharyngitis cases are caused by viruses, they are often (24%–70%) treated with antibiotics [5–7], given the difficulty of distinguishing between viral and bacterial pharyngitis. Clinical scores, most notably the Centor and McIsaac scores, are widely utilized to support the diagnosis of StrepA pharyngitis and decisions on antibiotic therapy. However, both scores have been demonstrated to have limited sensitivity [8].

Point-of-care tests (POCTs) were developed to test for StrepA in pharyngeal swabs. StrepA POCTs can provide rapid results in <15 minutes and do not require specialized laboratory equipment or training, allowing for timely decisions regarding treatment [9]. However, the sensitivity of StrepA POCTs is often not optimal, ranging from 50% to 95%, while the specificity is typically >90% [10, 11]. Clinical scores, such as the Centor or McIsaac score, and StrepA POCTs are often combined. Clinical scores are first used to estimate the likelihood of StrepA pharyngitis and determine whether a Strep A POCT is needed—typically testing is reserved for intermediate to high scores (eg, ≥2). The majority of available StrepA POCTs detect the Lancefield group A carbohydrate, a StrepA-specific cell-wall antigen. In recent years, molecular (nucleic acid amplification tests) tests have been developed and introduced to the market with very high sensitivity but relatively high costs [12].

There are conflicting reports on the effectiveness of StrepA POCTs to improve antibiotic prescribing and patient health outcomes in patients with symptoms of pharyngitis treated in outpatient settings. The objective of our systematic review is to identify available randomized controlled trials (RCTs) and to summarize the evidence on the impact of StrepA POCTs on antibiotic prescribing, clinical decisions, and patient health outcomes.

## METHODS

This study followed the guidelines from the Preferred Reporting Items for Systematic Reviews and Meta-Analyses (PRISMA) statement [13]. This study is part of a larger project of systematic reviews on the impact of POCT for pathogen identification on clinical decisions and patient health outcomes funded by the German Federal Ministry of Education and Research (grant number 01KG2315). The study protocol was published a priori in the Prospective Register for Systematic Reviews (PROSPERO, CRD42024539660) [14].

### Study Outcomes

The primary outcomes of this study were the impact of antigen or molecular StrepA POCTs on (*i*) antibiotic prescription, (*ii*) clinical decisions, and (*iii*) patient health (eg, symptom duration, days until pain resolution, follow-up healthcare visits, mortality). The secondary endpoint was the diagnostic accuracy of POCT for StrepA (eg, sensitivity, specificity).

### Search Strategy

We conducted a systematic search in Medline (via PubMed), Web of Science, Scopus, and the Cochrane Central Register of Controlled Trials (CENTRAL) for studies published after December 1999. The search was performed on 23 April 2024 and was updated on 8 January 2025. We established a sensitivity-maximizing search string including synonyms for point-of-care testing, outpatient setting, and randomized controlled trials. The complete search strategies for all databases, registers and websites, including any filters and limits used, are described in the Supplementary material. In addition, we checked the references of the included articles and performed a hand search.

### Study Selection

Articles were included if they met the following criteria:

- Study type: Individually or cluster-randomized controlled clinical trial (RCT).
- Participants: Patients of all ages with symptoms or a diagnosis of an acute respiratory tract infection (pharyngitis, cough, sore throat, etc).
- Intervention: POCTs for identification of StrepA (antigen detection or molecular testing), either alone or in combination with other interventions.
- Control: Standard care as defined in the studies.
- Outcomes: The study reported data on predefined outcomes.
- Setting: The study was performed in outpatient care settings, such as primary care practices and emergency departments.
- Publication date: The article was published after December 1999.
- Language: English, French, German, Portuguese, and Spanish.

At least 2 review authors (C. K.-S., A.-S. M., R. M.) independently assessed the titles, abstracts, and full texts for eligibility using COVIDence software [15]. Disagreements were resolved through discussion.

### Data Extraction and Risk of Bias Assessment

The data of all eligible studies were independently extracted by at least 2 reviewer authors (C. K.-S., A.-S. M., R. M.) using a standard tabulator form in Microsoft Excel. All disagreements were resolved through discussion. The following data were extracted: study identifier, publication year, name of the first author, study period, RCT design, study location, patient characteristics (age groups, infection, and comorbidities), intervention characteristics (POCT type [antigen or molecular test]), POCT manufacturer, and outcome measures. At least 2 review authors (C. K.-S., A.-S. M., R. M.) independently assessed risk of bias of the included studies using the Cochrane risk of bias tool [16]. The overall certainty of the evidence for the number of antibiotic prescriptions was assessed using the Grading of Recommendations, Assessment, Development, and Evaluations (GRADE) framework [17].

### Data Synthesis and Statistical Analysis

Only published data were used. When possible, we calculated a weighted estimate for the predefined outcomes by the means of a random-effects meta-analysis using the inverse variance method and the DerSimonian-Laird estimator for τ^2^. All statistical analyses were performed using R software version 4.1.2 [18] and the “meta” package [19]. Dichotomous data (eg, antibiotic prescription) were calculated as risk ratios (RRs) with 95% confidence intervals (CIs). Outcomes reported as continuous data (eg, reported medians or means for duration of symptoms) were presented narratively and in tabulator form.

We assessed statistical heterogeneity using the *I*^2^ statistic and performed a publication bias analysis for the primary outcome antibiotic prescription by the means of a funnel plot analysis.

The following predefined subgroup analyses were performed: age group (mixed-age population, children [0–17 years], adults [≥18 years]), RCT design (cluster randomization, individually randomized RCT), healthcare setting (pediatrician/primary care practices, emergency department), and StrepA POCT intervention (POCT manufacturer, StrepA POCT as the sole intervention vs StrepA POCTs combined with other interventions).

### Patient Involvement

We established an advisory board with 6 patients or patient representatives for this study. Patients were approached through a social media announcement. The perspectives and advices of patients and public representatives were included in all steps of the systematic review from the design of the search strategy to the interpretation of the results. More details on patients involvement are provided in the Supplementary material.

## RESULTS

**Figure 1.**
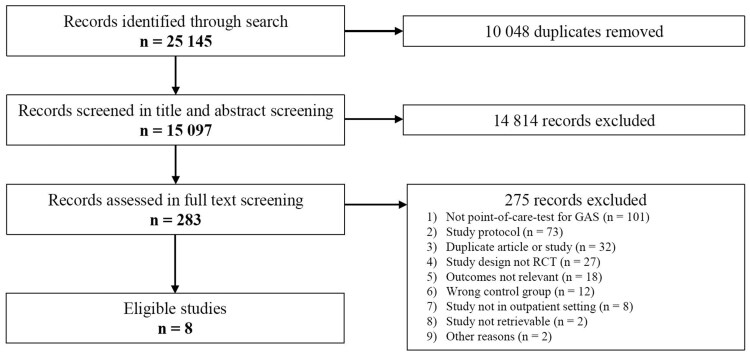
Study selection flowchart. Abbreviations: StrepA, group A β-hemolytic *Streptococcus*; RCT, randomized controlled trial.

Our systematic literature search yielded 15 097 unique records. After title, abstract and full-text screening, 8 studies were included in this systematic review (Figure 1) [20–27]. The studies were conducted between 2007 and 2021. Seven studies were conducted in pediatric or primary care practices and investigated antigen StrepA POCTs, while 1 study [20] was carried out in a pediatric emergency department using a molecular StrepA POCT (see Table 1 for full study characteristics). Five RCTs studied StrepA POCTs as the sole intervention and 3 studies investigated StrepA POCTs in combination with other interventions. All studies were conducted in Europe (n = 6) or Canada (n = 2).

**Table 1.** Study Characteristics of Included Studies

Study	RCT Design, Data Collection Period	Healthcare Setting, Study Location	Patients	Intervention (POCT)	Control (Standard Care)
Gill, 2024 [20]	Single-center RCT, 2019–2021	Pediatric emergency department, Canada	227 children (3–17 y) Patients with sore throat, with or without fever, and in whom the pediatrics clinician planned to collect a throat swab to culture based on their clinical judgment for suspected StrepA pharyngitis	Molecular StrepA POCT (ID NOW Strep A2, Abbott); results are communicated to the attending physician and participant family; if positive, the participant was prescribed antibiotics; if negative, the participant was not provided an antibiotic prescription	Throat swab and culture; patients were discharged with prescriptions for antibiotics that they were instructed to fill if contacted by a physician with a positive test result once available; in the event that a prescription was not provided before discharge, the physician contacting the family with a positive result would provide a prescription directly to the pharmacy
Wächtler, 2023 [21]	Cluster RCT, 2010–2012	61 primary care practices in Germany	520 children and adults (≥2 y) Patients with moderate to severe sore throat (≤2 wk)	Antigen StrepA POCT (TestPack Plus Strep A test, Alere) for patients with McIsaac score ≥3 + Implementation of guideline (“sore throat”); guidelines provided recommendations, but ultimately the GPs made their own decision on treatment	Standard clinical routine as established in practices; no treatment recommendations
Bonet-Esteve, 2021 [22]	Multicenter RCT, 2010–2011	27 pediatric practices in Spain	519 children (3–15 y) Symptoms indicating tonsillopharyngitis	Antigen StrepA POCT (test not reported) in addition to clinical diagnosis (Centor score); no treatment recommendations	Standard clinical routine as established in practices; no treatment recommendations
Małecki, 2017 [23]	Cluster RCT, 2013–2014	68 primary care practices in Poland	1307 children (2–15 y) Symptoms: pain, irritation or scratching of throat and/or tonsil) for 3 d; signs: redness and/or swelling and/or petechiae of the mucous membrane of throat and/or tonsils	Antigen StrepA POCT (OSOM Strep A test, Sekisui Diagnostics); no treatment recommendations	Anamnesis and clinical examination; no treatment recommendations
Little, 2013 [24]	Multicenter RCT, 2008–2011	Primary care practices in the United Kingdom	631 children and adults (≥3 y) Acute sore throat (≤2 wk of sore throat) and an abnormal-looking throat (erythema and/or pus)	Antigen StrepA POCT (test not reported) + Clinical score (FeverPAIN) to identify streptococcal infection; patients with low clinical scores (0/1) were not offered antibiotics or a rapid antigen test, those with a score of 2 were offered a delayed prescription, and those with higher scores (≥3) underwent a rapid antigen test; patients with negative test results were not offered antibiotics	Clinical score (FeverPAIN); antibiotics were not offered to those with low scores (0/1); immediate antibiotics were offered for those with high scores (≥4) and delayed antibiotics for those with intermediate scores (2/3)
Llor, 2011 [25]	Cluster RCT, 2008	20 primary care centers in Spain	543 adults (14–60 y) Diagnosed with acute pharyngitis with 1 or more Centor criteria: fever, sore throat, tonsillar exudate, tender cervical nodes, and/or absence of cough	Antigen StrepA POCT (OSOM Strep A test, Genzyme), no treatment recommendations	Clinical examination; no treatment recommendations
Maltezou, 2008 [26]	Cluster RCT, 2005–2007	17 private pediatrician practices in Greece	928 children (2–14 y) Clinical evidence of pharyngitis (at least 1 of the following criteria: fever [>38.0°C], tonsillar exudate, tender, enlarged, anterior cervical lymph nodes and absence of cough)	Antigen StrepA POCT (Link 2 Strep A Rapid Test, Becton-Dickinson) + Standard culture (results available after 48 h); antibiotic prescription only if positive POCT result; if positive culture, but RADT-negative, prescription of antibiotics was done 48 h later	Clinical examination; no treatment recommendations
Worrall, 2007 [27]	Cluster RCT, 2005	37 primary care practices in Canada	533 adults (≥19 y) Acute sore throat as primary symptom	Antigen StrepA POCT (Clearview Exact Strep A dipstick, Wampole Laboratories); no treatment recommendations	Standard clinical routine as established in practices; no treatment recommendations

Abbreviations: GP, general practitioner; POCT, point-of-care test; RADT, rapid antigen detection test; RCT, randomized controlled trial; StrepA, group A β-hemolytic *Streptococcus*.

**Figure 2.**
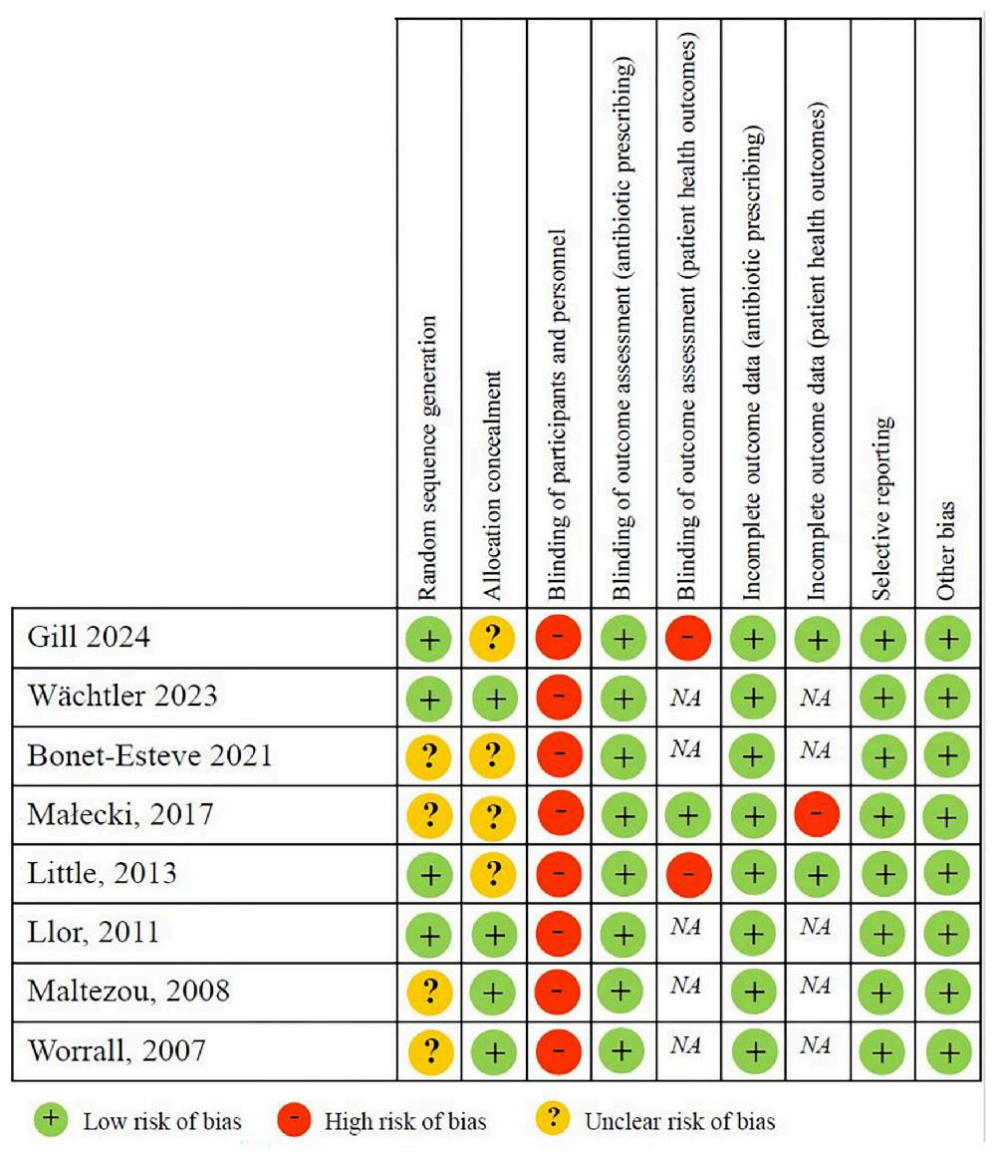
Risk of bias assessment using the Cochrane risk of bias tool. Abbreviation: NA, not applicable.

We assessed the risk of bias for each study and individually for the outcomes antibiotic prescribing and patient health outcomes. The risk of bias assessment is presented in Figure 2. None of the studies was blinded and random sequence generation as well as allocation concealment was unclear in 4 of the 8 studies. Overall, the studies had a low to moderate risk of bias.

The funnel plot analysis for the primary outcome of number of participants given an antibiotic prescription shows a symmetrical shape indicating no publication bias (Supplementary Figure 1. Supplementary Material).

### Antibiotic Prescribing

**Figure 3.**
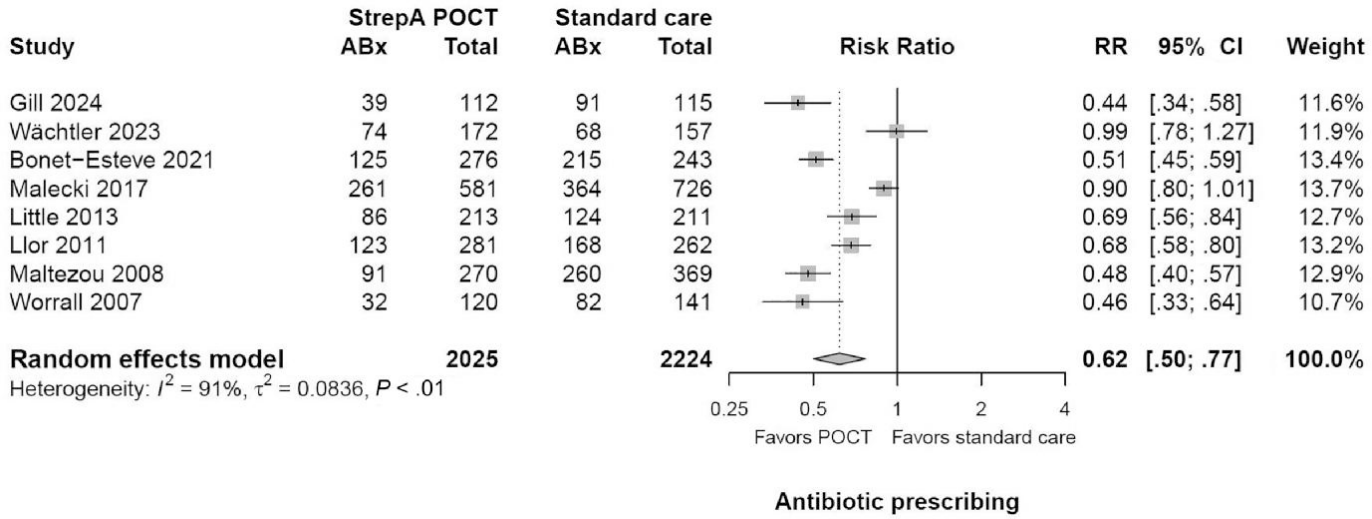
Forest plot of the impact of point-of-care testing for group A *Streptococcus* on the number of antibiotics prescribed. Abbreviations: ABx, antibiotics prescribed; CI, confidence interval; RR, risk ratio; StrepA POCT, point-of-care test for group A β-hemolytic *Streptococcus*.

All 8 studies, including 4249 participants, reported point estimates in favor of StrepA POCTs to reduce the number of participants given an antibiotic prescription (range of individual RRs, 0.44–0.99). The pooled result for all included trials shows that StrepA POCTs reduced antibiotic prescriptions by 38% in relation to standard care (RR, 0.62 [95% CI, .50–.77]; *P* < .0001) (Figure 3, Table 2).

**Table 2.** Summary of All Outcomes Assessed

Outcomes	No. of Participants (Studies)	Summary Effect Risk Ratio (95% CI, *P* Value)	Heterogeneity, *I*^2^ (*P* Value)
Antibiotic prescription			
Antibiotic prescribing	4249 (8 RCTs)	0.62 (.50–.77, *P* < .0001)	90.8% (*P* < .0001)
Inappropriate antibiotic prescribing	753 (2 RCTs [20, 25])	0.55 (.31–.97, *P* = .0375)	60.6% (*P* < .1110)
Other clinical decisions			
Ancillary testing	227 (1 RCT [20])	0.98 (.77–1.25, *P* = .8522)	…
Other therapeutic interventions	227 (1 RCT [20])	0.90 (.71–1.15, *P* = .3977)	…
Patient health outcomes			
Follow-up healthcare visits	1744 (3 RCTs [20, 23, 24])	0.56 (.29–1.09, *P* = .0863)	87.1% (*P* = .0004)
Days until pain resolution, mean	227 (1 RCT [20])	StrepA POCT: 3.9 d	…
		Standard care: 3.9 d	
Duration of symptoms rated as moderately bad or worse, d, median	325 (1 RCT [24])	StrepA POCT: 4 d	…
		Standard care: 5 d	
Mean of severity of sore throat and difficulty swallowing on days 2–4 (7-point scale: 0 = no problem, 6 as bad as it could be):	325 (1 RCT [24])	StrepA POCT: 2.83	…
		Standard care: 3.11	
Days until fever resolution, mean	227 (1 RCT [20])	StrepA POCT: 1.9 d	…
		Standard care: 2.2 d	
Days of school missed	227 (1 RCT [20])	StrepA POCT: 1.7 d	…
		Standard care: 1.9 d	
Days of work missed	227 (1 RCT [20])	StrepA POCT: 1.2 d	…
		Standard care: 1.4 d	
Length of stay			
Length of stay in emergency department, min, mean	227 (1 RCT [20])	StrepA POCT: 248 min	…
		Standard care: 245 min	

Abbreviations: CI, confidence interval; POCT, point-of-care test; RCT, randomized controlled trial; StrepA, group A β-hemolytic *Streptococcus*.

There was a considerable heterogeneity (*I*^2^ = 91%) that could not be explained by our preplanned subgroup analyses (Supplementary Table 1, Supplementary Material). Subgroup analyses indicate that StrepA POCTs decreased the number of antibiotics prescribed in children (RR, 0.56 [95% CI, .39–.81]; *P* = .002; 4 RCTs) and adults (RR, 0.57 [95% CI, .39–.85]; *P* = .006; 2 RCTs). Moreover, RCTs investigating the effects of StrepA POCTs without any other intervention show that StrepA POCTs reduced antibiotic prescriptions by 41% (RR, 0.59 [95% CI, .44–.78]; *P* < .001; 5 RCTs). Antigen StrepA POCTs were shown to be effective to decrease the number of antibiotic prescriptions in primary care practice settings (RR, 0.65 [95% CI, .53–.81]; *P* < .001; 7 RCTs). One study conducted using a molecular POCT for StrepA detection indicates a reduction of antibiotic prescription by 56% in a pediatric emergency department (RR, 0.44 [95% CI, .34–.57]; *P* < .001).

Two RCTs with 753 participants investigated the effects of StrepA POCTs on the number of inappropriate antibiotic prescriptions (Table 2). The pooled result shows that StrepA POCTs reduced inappropriate antibiotic prescriptions by 45% in relation to standard care (RR, 0.45 [95% CI, .31–.97]; *P* = .038).

### Other Clinical Decisions

The study by Gill et al [20] carried out in a pediatric emergency department showed that the use of a molecular POCT for StrepA detection did not reduce the number of ancillary testing (RR, 0.98 [95% CI, .77–1.25]; *P* = .852) and other therapeutic interventions, such as analgesics (RR, 0.90 [95% CI, .71–1.15]; *P* = .398) (Table 2).

### Patient Health Outcomes

Three RCTs reported different patient health outcomes, such as follow-up healthcare visits (3 RCTs), days until symptom/pain resolution (2 RCTs), severity of sore throat on days 2–4 (1 RCT), and days of school/work missed (1 RCT) (Table 2). The point estimates of the individual studies did not show differences between the use of StrepA POCTs and standard care in terms of health outcomes. Three RCTs with 1744 participants showed that there is a nonstatistical significant trend, that StrepA POCTs reduce follow-up health visits compared to standard care (RR, 0.56 [95% CI, .29–1.09]; *P* = .086) (Table 2). No study provided data on mortality.

### Diagnostic Accuracy

Three studies reported diagnostic accuracy data for antigen StrepA POCT (Table 3). StrepA prevalence in patients with signs of pharyngitis were between 17% and 32%. Reported sensitivity and specificity of StrepA POCTs ranged between 65% and 90%, and 85% and 94%, respectively. Positive predictive values were between 46% and 82%, while the negative predictive values ranged between 92% and 98%.

**Table 3.** Diagnostic Accuracy of Antigen Point-of-Care Test for Group A *Streptococcus*

Study	Prevalence of Group A *Streptococcus* in Pharyngitis	Sensitivity	Specificity	PPV	NPV
Wächtler, 2023 [21]	17%	65%	85%	46%	92%
Llor, 2011 [25]	18%	90%	94%	76%	98%
Maltezou, 2008 [26]	32%	83%	93%	82%	94%

In all studies, standard laboratory culture of pharyngeal swabs for group A *Streptococcus* was the gold standard.

Abbreviations: NPV, negative predictive value; PPV, positive predictive value.

## DISCUSSION

We identified 8 RCTs from North America and Europe. Five RCTs studied StrepA POCTs as the sole intervention and 3 studies investigated StrepA POCTs in combination with other interventions. Point-of-care testing for StrepA in children and adults with pharyngitis likely reduces the number of participants given an antibiotic prescription. StrepA POCTs may also reduce inappropriate antibiotic prescriptions and are also unlikely to affect patient health outcomes, regarding recovery. Overall, the included studies were at low to moderate risk of bias. The overall certainty of the evidence for the number of antibiotic prescriptions was moderate to high according to the GRADE assessment. Further clinical studies are unlikely to alter our overall conclusion on antibiotic prescribing, but may change the effect sizes due to the heterogeneity observed in our study.

Our pooled estimates indicate that the use of StrepA POCTs in patients with pharyngitis can reduce the number of prescribed antibiotics by 38% and in studies with StrepA POCTs as the sole intervention by 41%. Given the high incidence of pharyngitis in outpatient care and the increasing relevance of antibiotic resistance, this substantial reduction of antibiotic prescribing is an important antibiotic stewardship measure to preserve the effectiveness of antibiotics. However, the precise effect estimates should be interpreted with caution due to the considerable heterogeneity observed in our analysis that could not be explained by our subgroup analyses.

Sources of heterogeneity might be differences in inclusion/exclusion criteria, variations in local guidelines regarding StrepA diagnosis and therapy, application of different StrepA POCTs with different sensitivity, and local prevalence of StrepA in pharyngitis, as well as physicians’ and patients’ attitudes toward antibiotic use for sore throat.

In addition to StrepA POCTs, other POCTs have been shown to be effective in safely reducing the number of prescribed antibiotics in acute respiratory infections in outpatient care, such as the biomarkers C-reactive protein [28] and procalcitonin [29]. On the other hand, POCT for influenza and rapid multiplex polymerase chain reaction testing for respiratory viruses do not decrease antibiotic prescribing [30, 31].

Although our results indicate that StrepA POCTs can safely reduce the number of antibiotics and inappropriate antibiotic prescriptions in adults and children with pharyngitis, their utilization in primary care differs between countries. While StrepA POCTs are commonly employed in primary care in Scandinavian countries [32, 33] and the United States [34], they are not frequently used in Belgium, The Netherlands, or the United Kingdom [35]. In Germany, only 24% of general practitioners utilize StrepA POCTs but its use is higher among pediatricians [36, 37]. Barriers of StrepA POCT utilization might be inability to differentiate carriage from active infection, costs, inadequate reimbursement, accessibility, time constraints, and limited sensitivity [38–40]. Depending on the prevalence of StrepA pharyngitis in the analyzed RCTs, positive predictive values of antigen StrepA POCTs were 46%–82%, which means the validity of positive test results is relatively low. On the other hand, the negative predictive values were >90%, which means that negative antigen StrepA POCT results can exclude a StrepA pharyngitis and, therefore, can support decision against antibiotics. Molecular StrepA POCTs have been shown to have a sensitivity of >90% and almost 100% specificity [10].

One important reason to treat StrepA pharyngitis with antibiotics is the prevention of clinical complications. These include the development of acute rheumatic fever, peritonsillar abscesses, otitis media, and the transmission of StrepA. However, none of these outcomes were investigated in the included studies, likely due to the difficulties to measuring these outcomes in RCTs. Importantly, in high-income countries, the most clinically relevant adverse outcome of StrepA pharyngitis, acute rheumatic fever, has become exceedingly rare. Moreover, the majority of cases of (StrepA) pharyngitis are self-limiting in otherwise healthy patients and antibiotics do not necessarily result in substantial health benefits. These facts also support a prudent use of antibiotics.

It is important to note that in the case of positive StrepA test results, it is difficult to determine whether StrepA is the causative organism or a colonizer in patients with pharyngitis. Clinical scores, such as the Centor and McIsaac scores, can help to estimate the likelihood of StrepA-induced pharyngitis and can therefore guide the application and interpretation of StrepA POCTs as wells decisions on antibiotic use. Importantly, pharyngitis may also be caused by other pathogens, including *Streptococcus pneumoniae* and *Haemophilus influenzae*, which may require antibiotic treatment. Physicians should therefore not rely exclusively on StrepA test results, but should also integrate anamnesis, clinical signs and, ideally, other microbiological test results when deciding whether or not to prescribe antibiotics.

### Strengths and Limitations

To our knowledge, our study represents the first systematic review and meta-analysis of RCTs on the effects of StrepA POCTs on antibiotic prescription and patient health outcomes in patients with pharyngitis in outpatient care settings. However, our study has limitations. The statistical heterogeneity in the conducted meta-analyses is large, which limits the precision of the pooled estimates sizes. Three RCTs reported various patient health outcomes, which prevented conducting meta-analyses to quantify the impact of StrepA POCTs on patient health outcomes, such as recovery time. However, all individual point estimates indicate that the use of StrepA POCTs does not negatively impact patient health outcomes. None of the RCTs were blinded to patients and study personnel, which introduces a potential risk of bias, especially in the assessment of subjective outcomes, such as pain and symptom resolution.

## CONCLUSIONS

The use of POCTs for group A β-hemolytic *Streptococcus*, in addition to taking a medical history and clinical examination in children and adults with signs of pharyngitis, likely reduces the number of participants given an antibiotic prescription. Physicians can therefore use StrepA POCTs to inform their decisions on antibiotic prescribing without compromising patient health outcomes. In clinical practice, physicians must be aware of the limitations of StrepA POCTs, in particularly the limited sensitivity for detecting StrepA.

## Supplementary Material

ofaf407_Supplementary_Data

## Supplementary Data

Supplementary materials are available at *Open Forum Infectious Diseases* online. Consisting of data provided by the authors to benefit the reader, the posted materials are not copyedited and are the sole responsibility of the authors, so questions or comments should be addressed to the corresponding author.
