# Supplementary material for: The Impact of Point-of-Care Testing for Group A Streptococcal Pharyngitis on Antibiotic Prescribing and Patient Health Outcomes in Outpatient Care: A Systematic Review and Meta-analysis of Randomized Controlled Trials
Source: Open Forum Infect Dis. 2025 Jul 9;12(7):ofaf407. doi: 10.1093/ofid/ofaf407 (PMC12301963; doi:10.1093/ofid/ofaf407)
Supplement: ofaf407_Supplementary_Data [file ofaf407_supplementary_data.docx]

**- Supplementary Material -**

**The impact of Point-of-care testing for Group A streptococcal pharyngitis on antibiotic prescribing and patient health outcomes in outpatient care: a systematic review and meta-analysis of randomized controlled trials**

Ann-Sophie Mägdefrau^*^ ^1, 2^, Carolin Kathner-Schaffert^* 1^, Anni Matthes^1, 2^, Jutta Bleidorn^1^ and Robby Markwart^1, 2^

^1^ Institute of General Practice and Family Medicine, Jena University Hospital, Friedrich Schiller University Jena, Bachstrasse 18, 07743 Jena, Germany

^2^ InfectoGnostics Research Campus Jena, Philosophenweg 7, 07743, Jena, Germany.

* These authors contributed equally

^#^Corresponding Author:

**Robby Markwart**

[robby.markwart@med.uni-jena.de](mailto:robby.markwart@med.uni-jena.de)

+49(0)3641-9395820

Table of Contents

[Patient involvement 3](#_Toc191470422)

[Search strings 4](#_Toc191470423)

[Funnel plot 7](#_Toc191470424)

[Subgroup analyses for antibiotic prescribing and follow-up visits to healthcare 8](#_Toc191470425)

# Patient involvement

We established an advisory board with six patients or patient representatives for this study. Patients were approached through a social media announcement. We assembled a group aiming for diversity with regard to sex (three female, three males), age (18 to 59 years), educational and cultural background (heterogeneous educational backgrounds, one person with a migration background) and experience of illness. The patients actively contributed to the identification of relevant research questions (e.g. special focus on point-of-care diagnostics in paediatric settings), outcomes (e.g. psychological wellbeing, prevention of infection transmission) as well as inclusion and exclusion criteria (inclusion of hospital outpatient clinics). Subsequently, the patients actively contributed to the identification of patient-relevant findings (e.g. individual health effects, public health effects) and their interpretation based on real-world experiences. With regard to the dissemination of results, patients contributed to the translation of findings into patient-friendly summaries (e.g. headline ‘No more unnecessary antibiotics!’ instead of ‘The impact of point-of-care testing for …’), presenting at a POCT symposium (presentation by one patient representative) and input where results could be shared (e.g. local health week). Three meetings, each lasting two hours, with a 100% participation rate took place. Patients received remuneration of 75€ for each meeting attended.

# Search strings

***MEDLINE (via Pubmed)***

Conducted on 24 April 2024 and updated on 8 January 2025

("primary care"[All Fields] OR "primary healthcare"[All Fields] OR "primary health care"[All Fields] OR "primary physician"[All Fields] OR "primary practitioner"[All Fields] OR "ambulatory care"[All Fields] OR "ambulatory visit*"[All Fields] OR "ambulatory practice"[All Fields] OR "ambulatory healthcare"[All Fields] OR "ambulatory health care"[All Fields] OR "emergency care"[All Fields] OR "emergency department"[All Fields] OR "emergency room"[All Fields] OR "outpatient care"[All Fields] OR "outpatient healthcare"[All Fields] OR "outpatient health care"[All Fields] OR "outpatient treatment"[All Fields] OR "day patient care"[All Fields] OR "general care"[All Fields] OR "general healthcare"[All Fields] OR "general health care"[All Fields] OR "general physician*"[All Fields] OR "general practi*"[All Fields] OR "primary physician*"[All Fields] OR "gynaecolog"[All Fields] OR "gynecolog"[All Fields] OR "family doctor*"[All Fields] OR "family physician*"[All Fields] OR "family practi*"[All Fields] OR "paediatri*"[All Fields] OR "pediatri*"[All Fields] OR "urolog*"[All Fields] OR "ENT"[All Fields] OR "Ear Nose Throat"[All Fields] OR "ear nose and throat"[All Fields] OR "obstetric*"[All Fields] OR "genitourinary*"[All Fields] OR "otolaryngolog*"[All Fields] OR "rhinolaryngolog*"[All Fields] OR "nursery home"[All Fields] OR "nursing home"[All Fields] OR "residential care"[All Fields] OR "care home"[All Fields])

AND

("POCT"[All Fields] OR "POC"[All Fields] OR "point of care"[All Fields] OR "bedside"[All Fields] OR "bed side"[All Fields] OR "on site"[All Fields] OR "near patient"[All Fields] OR "strip test*"[All Fields] OR "rapid diagnos*"[All Fields] OR "rapid test*"[All Fields] OR "rapid measur*"[All Fields] OR "rapid device*"[All Fields] OR "mobile test*"[All Fields] OR "mobile diagnos*"[All Fields] OR "mobile measur*"[All Fields] OR "non laboratory based"[All Fields] OR "lateral flow"[All Fields] OR "rapid antigen"[All Fields] OR "RDT"[All Fields] OR "RDTs"[All Fields] OR "semiquantitative test*"[All Fields] OR "test strip"[All Fields])

AND

("randomized controlled trial"[All Fields] OR "randomised controlled trial"[All Fields] OR "controlled clinical trial"[All Fields] OR "clinical trials as topic"[All Fields] OR "trial"[All Fields] OR "control group*"[All Fields] OR "group*"[All Fields] OR "rct"[All Fields] OR "rcts"[All Fields] OR "randomi*"[All Fields] OR "random assignment"[All Fields] OR "crossover design"[All Fields] OR "comparative stud*"[All Fields] OR "single blind stud*"[All Fields] OR "double blind stud*"[All Fields] OR "cluster rct"[All Fields] OR "cluster rcts"[All Fields] OR "placeboes"[All Fields] OR "placebos"[MeSH Terms] OR "placebos"[All Fields] OR "placebo"[All Fields])

AND

(2000:2025[pdat])

***Web of Science***

Conducted on 24 April 2024 and updated on 8 January 2025

(ALL=("primary care" OR "primary healthcare" OR "primary health care" OR "primary physician" OR "primary practitioner" OR "ambulatory care" OR "ambulatory visit*" OR "ambulatory practice" OR "ambulatory healthcare" OR "ambulatory health care" OR "emergency care" OR "emergency department" OR "emergency room" OR "outpatient care" OR "outpatient healthcare" OR "outpatient health care" OR "outpatient treatment" OR "day patient care" OR "general care" OR "general healthcare" OR "general health care" OR "general physician*" OR "general practi*" OR "primary physician*" OR "gynaecolog" OR "gynecolog" OR "family doctor*" OR "family physician*" OR "family practi*" OR "paediatri*" OR "pediatri*" OR "urolog*" OR "ENT" OR "Ear Nose Throat" OR "ear nose and throat" OR "obstetric*" OR "genitourinary*" OR "otolaryngolog*" OR "rhinolaryngolog*" OR "nursery home" OR "nursing home" OR "residential care" OR "care home")) AND (ALL=("POCT" OR "POC" OR "point of care" OR "bedside" OR "bed side" OR "on site" OR "near patient" OR "strip test*" OR "rapid diagnos*" OR "rapid test*" OR "rapid measur*" OR "rapid device*" OR "mobile test*" OR "mobile diagnos*" OR "mobile measur*" OR "non laboratory based" OR "lateral flow" OR "rapid antigen" OR "RDT" OR "RDTs" OR "semiquantitative test*" OR "test strip")) AND (ALL= ("randomized controlled trial" OR "randomised controlled trial" OR "controlled clinical trial" OR "clinical trials as topic” OR "trial" OR "control group*" OR "group*" OR "rct" OR "rcts" OR "randomi*" OR "random assignment" OR "crossover design" OR "comparative stud*" OR "single blind stud*" OR "double blind stud*" OR "cluster rct" OR "cluster rcts" OR "placeboes" OR "placebos" OR "placebo")) AND (PY=(2000-2025))

***Cochrane Central Register of Controlled Trials (CENTRAL)***

Conducted on 24 April 2024 and updated on 8 January 2025

Fields: Title, Abstract, Keywords; Filter: 2000 – 2025

(primary NEXT care OR primary NEXT healthcare OR primary NEXT health NEXT care OR primary NEXT physician OR primary NEXT practitioner OR ambulatory NEXT care OR ambulatory NEXT visit* OR ambulatory NEXT practice OR ambulatory NEXT healthcare OR ambulatory NEXT health NEXT care OR emergency NEXT care OR emergency NEXT department OR emergency NEXT room OR outpatient NEXT care OR outpatient NEXT healthcare OR outpatient NEXT health NEXT care OR outpatient NEXT treatment OR day NEXT patient NEXT care OR general NEXT care OR general NEXT healthcare OR general NEXT health care OR general NEXT physician* OR general NEXT practi* OR primary NEXT physician* OR gynaecolog OR gynecolog OR family NEXT doctor* OR family NEXT physician* OR family NEXT practi* OR paediatri* OR pediatri* OR urolog* OR ENT OR Ear NEXT Nose NEAR Throat OR obstetric OR genitourinary* OR otolaryngolog* OR rhinolaryngolog* OR nursery NEAR home OR nursing NEAR home OR residential NEAR care OR care NEAR home OR home NEXT care OR home NEXT based)

AND

(POCT OR POC OR point NEXT of NEXT care OR point NEXT of NEXT care NEAR test OR bedside OR bed NEXT side OR onsite OR “near patient” OR strip NEXT test OR rapid NEXT diagnos* OR rapid NEXT test* OR rapid NEXT measur* OR rapid NEXT device* OR Mobile NEXT test* OR mobile NEXT diagnos* OR mobile NEXT measur* OR non NEXT laboratory NEXT based OR lateral NEXT flow OR rapid NEXT antigen OR RDT OR RDTs OR semiquantitative NEXT test* OR test strip)

***Scopus***

Conducted on 24 April 2024 and updated on 8 January 2025

TITLE-ABS-KEY ( ( "POCT" OR "POC" OR "point of care" OR "bedside" OR "bed side" OR "on site" OR "near patient" OR "strip test*" OR "rapid diagnos*" OR "rapid test*" OR "rapid measur*" OR "rapid device*" OR ( rapid W/5 diagnos* ) OR ( rapid W/5 test* ) OR ( rapid W/5 measur* ) OR ( rapid W/5 device* ) OR "mobile test*" OR "mobile diagnos*" OR "mobile measur*" OR "non laboratory based" OR "lateral flow" OR "rapid antigen" OR "RDT" OR "RDTs" OR "semiquantitative test*" OR "test strip" )

AND

( "primary care" OR "primary healthcare" OR "primary health care" OR "primary physician" OR "primary practitioner" OR "ambulatory care" OR "ambulatory visit*" OR "ambulatory practice" OR "ambulatory healthcare" OR "ambulatory health care" OR "emergency care" OR "emergency department" OR "emergency room" OR "outpatient care" OR "outpatient healthcare" OR "outpatient health care" OR "outpatient treatment" OR "day patient care" OR "general care" OR "general healthcare" OR "general health care" OR "general physician*" OR "general practi*" OR "primary physician*" OR "gynaecolog" OR "gynecolog" OR "family doctor*" OR "family physician*" OR "family practi*" OR "paediatri*" OR "pediatri*" OR "urolog*" OR "ENT" OR "Ear Nose Throat" OR "obstetric*" OR "genitourinary*" OR "otolaryngolog*" OR "rhinolaryngolog*" OR "nursery home" OR "nursing home" OR "residential care" OR "care home" OR "home care" OR "home-based" OR ( nose W/5 throat ) )

AND

( "randomized controlled trial" OR "randomised controlled trial" OR "controlled clinical trial" OR "clinical trials as topic" OR "trial" OR "control group*" OR "group*" OR "rct" OR "rcts" OR "randomi*" OR "random assignment" OR "crossover design" OR "comparative stud*" OR "single blind stud*" OR "double blind stud*" OR "cluster rct" OR "cluster rcts" OR "placeboes" OR "placebos" OR "placebos" OR "placebo" ) )

AND

( PUBYEAR > 1999 AND PUBYEAR < 2026 )

# Funnel plot

**sFigure 1.** Funnel plot of risk ratios for antibiotic prescribing from all included trials


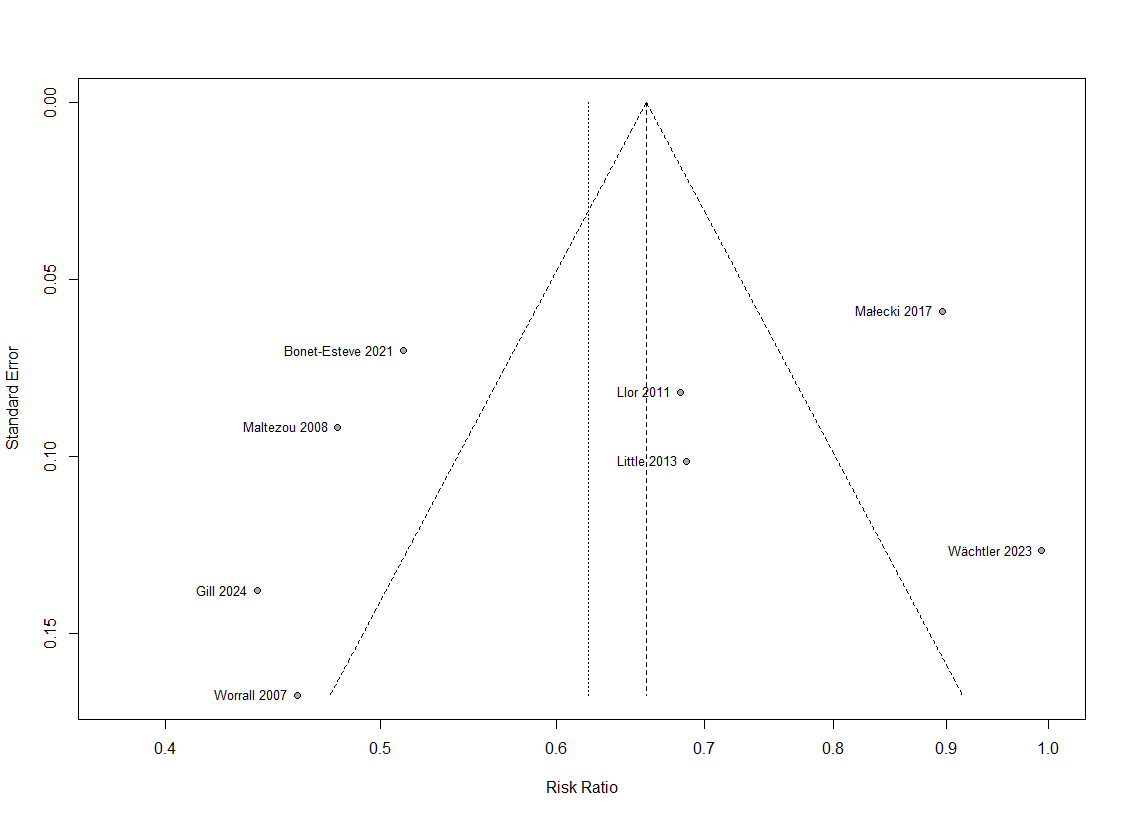


**Alt text for sFigure 1.** Graphic depicts a funnel plot to assess potential publication bias. Each dot represents an individual study with the risk ratio of antibiotic prescribing (point-of-care testing for Group A Streptococcus vs. Standard care) on the X-axis and the standard error on the Y-axis.

# Subgroup analyses for antibiotic prescribing and follow-up visits to healthcare

**sTable 1.** Subgroup analyses for the effects of point-of-care testing for Group A *Streptococcus* on antibiotic prescribing and follow-up visits to healthcare.

| **Outcomes** | **Subgroup** | **No. of participants (studies)** | **Summary effect**  **Risk Ratio**  **(95%CI, p-value)** | **Heterogeneity**  **(I^2^ in %, p-value)** |
| --- | --- | --- | --- | --- |
| ***Age group*** | | | | |
| Antibiotic prescribing | All age groups | 4,150  (8 RCTs) | **0.62**  (0.51 - 0.77, p < 0.0001) | 90.8 %  (p < 0.0001) |
|  | Adults | 804  (2 RCTs) | **0.57**  (0.39 - 0.85, p = 0.0056) | 78.7 %  (p = 0.0301) |
|  | Children | 2,692  (4 RCTs) | **0.56**  (0.39 - 0.81, p = 0.0018) | 95.0 %  (p = 0.0018) |
| Follow-up visit to healthcare | All age groups | 1,744  (3 RCTs) | **0.56**  (0.29 - 1.09, p = 0.0863) | 87.1 %  (p = 0.0004) |
|  | Children | 1,322  (2 RCTs) | **0.50**  (0.21 - 1.15, p = 0.1035) | 92.0 %  (p = 0.0004) |
| ***Intervention type*** | | | | |
| Antibiotic prescribing | GAS POCT alone | 2,857  (5 RCTs) | **0.59**  (0.44 - 0.78, p = 0.0002) | 92.5 %  (p < 0.0001) |
|  | GAS POCT + other intervention | 1,293  (3 RCTs) | **0.68**  (0.46 - 1.02, p = 0.0639) | 91.2 %  (p < 0.0001) |
| Follow-up visit to healthcare | GAS POCT alone | 1,322  (2 RCTs) | **0.50**  0.21 - 1.15, p = 0.1035 | 92.0 %  (p = 0.0004) |
|  | GAS POCT + other intervention | 422  (1 RCT) | **0.78**  (0.38 - 1.52, p = 0.4344) | - |
| ***Healthcare setting*** | | | | |
| Antibiotic prescribing | Primary care practices | 3,923  (7 RCTs) | **0.65**  (0.53 - 0.81, p = 0.0001) | 91.1 %  (p < 0.0001) |
|  | Emergency department | 227  (1 RCT) | **0.44**  (0.34 - 0.57, p < 0.001) | - |
| Follow-up visit to healthcare | Primary care practices | 1,517  (2 RCTs) | **0.47**  0.21 - 1.04, p = 0.0637 | 92.0 %  (p = 0.0004) |
|  | Emergency department | 227  (1 RCT) | **0.78**  (0.51- 1.18, p = 0.2349) | - |
| ***Randomization method*** | | | | |
| Antibiotic prescribing | Cluster randomization | 3,079  (5 RCTs) | **0.67**  (0.51 - 0.90, p = 0.0071) | 91.6 %  (p < 0.0001) |
|  | Individual randomization | 1,071  (3 RCTs) | **0.54**  (0.43 - 0.68, p < 0.001) | 76.3 %  (p = 0.0146) |
| Follow-up visit to healthcare | Cluster randomisation | 1,095  (1 RCT) | **0.33**  (0.26 - 0.41, p < 0.0001) | - |
|  | Individual randomization | 649  (2 RCTs) | **0.77**  (0.54 - 1.10, p = 0.1555) | 0 %  (p = 0.9506) |
